# Updates in MRI characterization of the thymus in myasthenic patients

**Published:** 2012-06-18

**Authors:** GA Popa, EM Preda, C Scheau, C Vilciu, IG Lupescu

**Affiliations:** * Department of Radiology and Medical Imaging, “Fundeni” Clinical Institute, Bucharest; ** Department of Neurology, “Fundeni” Clinical Institute, Bucharest

**Keywords:** myasthenia gravis, thymus, hyperplastic thymus, thymoma

## Abstract

**Purpose:** To evaluate the imaging appearance of the thymus in the myasthenic patients by using chemical shift magnetic resonance imaging, and, to correlate the chemical shift ratio (CSR) with pathologic findings after surgical excision.

**Materials and Methods:** In the past year, a total of 11 myasthenic patients (4 males, 7 females; age range of 26-65 years), have been investigated by MRI centered at the thymic lodge. Our protocol included a Dual-Echo technique, T1-weighted In-phase/Opposed-phase MR images in all patients. A chemical shift ratio (CSR) was calculated by comparing the signal intensity of the thymus gland with that of the chest wall muscle for quantitative analysis. For this purpose, we have used standard region-of-interest electronic cursors at a slice level of the maximum axial surface of the thymus. We have identified two patients groups: a thymic hyperplasia group and a thymic tumoral group.

**Results:** With the decrease in the signal intensity of the thymus gland at chemical shift, the MR imaging was evident only in the hyperplasia group. The mean CSR in the hyperplasia group was considerably lower than that in the tumor group, 0,4964 ± 0,1841, compared with 1,0398 ± 0,0244. The difference in CSR between the hyperplasia and tumor groups was statistically significant (P=0,0028).

**Conclusion:** MR imaging using T1-weighted In-phase/Opposed-phase images could be a useful diagnostic tool in the preoperative assessment of the thymic lodge and may help differentiate thymic hyperplasia from tumors of the thymus gland

**Abbreviations:** myasthenia gravis – MG; chemical shift ratio – CSR; frequency-encoding direction – FED

## Introduction

The examination of the thymus gland is usually done by computed tomography (CT). In the Department of Radiology and Medical Imaging of “Fundeni” Clinical Institute, this type of examination is performed mainly in patients with myasthenia gravis (MG), admitted in the Department of Neurology and in the Department of General Surgery and Liver Transplantation, with an experience of 30 years regarding CT examinations.

Sometimes, the CT appearance of the thymus, correlated with the patient's age do not allow a specific diagnosis, such as diffuse increase in size of the thymus, this appearance being seen in thymic hyperplasia, thymomas and reactive changes of the thymus frequently associated with chemotherapy, radiotherapy, corticosteroid therapy, and severe burns.

Minimal invasive surgical techniques including transcervical incision and video-assisted thoracoscopic surgery and robotic-assisted thymectomy are not recommended in patients with thymic malignancies and high risk of local recurrence. Therefore, the importance of preoperative evaluation of the thymic abnormalities has increased as more therapeutic techniques have been developed [**1**]. 

Chemical shift MR imaging with Dual-Echo sequences In-phase/Opposed-phase proved extremely useful in the diagnosis of the lesions with substantial fatty elements. Chemical Shift imaging can aid in the diagnosis of lipid-containing lesions of the brain (lipoma, dermoid, and teratoma) or the body such as adrenal adenoma, focal fat within the liver, and angiomyolipoma [**2**].

Therefore, we have come up with the following hypothesis, that MRI may be useful in identifying normal thymus in younger patients, thymic hyperplasia and thymic tumoral masses, when we used Dual-Echo sequences In-phase/Opposed-phase (Chemical-Shift MR Imaging).

## Objective

The aim of this prospective study is to assess the importance of a MRI examination in characterizing thymic lodge in myasthenic patients, especially in differentiating the thymic hyperplasia from tumors of the thymus gland, taking into consideration that the chemical-shift MR imaging with Dual-Echo In-phase/Opposed-phase sequences depicts no decrease in signal intensity of thymic tumors, unlike the decrease in signal intensity of the thymic hyperplasia, due to the fact that the thymic tumors do not have a lipid component included.

### Physical Basis of the Chemical Shift

When an external magnetic field is applied to the tissue, its nuclei will resonate at a specific frequency that depends on the strength of that external magnetic field (B0) and its local microenvironment. The effects of the external magnetic field can be determined by the Larmor equation: ω0 = γ • B0, where ω is the resonant or precessional frequency and γ is the gyromagnetic ratio for a specific nuclear species [**2**].

The Chemical Shift is a consequence of the variations in resonance frequency (Larmor frequency) of the protons located in different chemical environments. The effects of the local environment on the individual precessional frequency of each nucleus are further influenced by the electron shell interactions of each nucleus with those of surrounding molecules [**2**].

In clinical imaging, hydrogen protons form the basis for MR image signal and contrast. The chemical shift phenomenon is the most noticeable between the hydrogen protons of lipid and those of water, because of their large relative differences in magnetic shielding. The difference in resonant frequencies between lipid and water protons increases proportionately with the static magnetic field strength [**2**].

The approximate chemical shift between human lipid and water is of 3.5 parts per million. When taking into account a field strength of 1.5 T, this shift translates to approximately a 224-Hz separation between the resonant frequencies of fat and water protons [**2,3**].

**Fig. 1:**
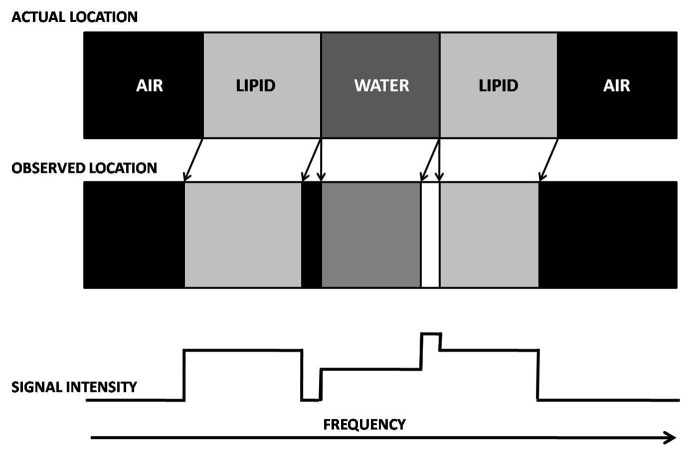
Schematic depicts chemical shift along the frequency-encoding direction. Lipid signal is shifted to a lower-frequency position when it is surrounded by water, which leaves a signal void (dark band) on the high-frequency side of the lipid structure and an increase in signal (bright band) on the low-frequency side [**2**].

This causes misregistration of the position of lipid proton voxels along the frequency-encoding direction (FED) with a positional shift toward the direction of decreasing frequencies. At a planar lipid-water interface, oriented perpendicularly to the FED, this positional misregistration can cause two effects. A linear band of pixels with decreased signal intensity occurs at the interface, when lipid is on the low-frequency side of the FED (with respect to water). This results from the lipid pixels that are shifted away from the water pixels, which leaves pixels on the lipid side of the interface with absent signal. A linear band of pixels with increased signal intensity occurs at the interface when lipid is on the high frequency side of the FED (with respect to water). This results from the lipid pixels being shifted toward the water pixels, which causes pixels on the waterside of the interface to have signal intensity equal to the sum of the lipid- and water-containing pixels [**4**]. (**Fig. 1**)

The chemical shift also depends on sampling the bandwidth. The distance of the chemical shift artifact is inversely proportional to the sampling bandwidth [**3**]. This corresponds to all frequencies contained in the signal, applying a gradient during signal reception, leading to frequency dispersion. Depending on the number of samples in the direction of frequency encoding (or the number of pixels in this direction) and the time of observation corresponds to the period of time the read gradient is applied.

## Materials and Methods

In the past year, a total of 11 myasthenic patients (4 males, 7 females; age range of 26-65 years), have been investigated by MRI centered at the thymic lodge, in the Department of Radiology and Medical Imaging of “Fundeni” Clinical Institute.

The patients were assigned to two groups: a hyperplasia group consisting of five patients with hyperplastic thymus and a tumor group consisting of six patients with thymoma. We obtained a pathologic proof of the diagnosis for all the patients in this study.

The mean ages of the patients in the hyperplasia and tumor groups were of 31 years ± 5.83 and 52.67 years ± 9.07, respectively. The patients in the hyperplasia group were significantly younger than those in the tumor group (P = 0.0014).

All MR imaging was performed by using a 1.5-T unit (General Electric Medical Systems Genesis Signa). A phased-array coil (Torsopa; GE Medical Systems) was also used. Imaging was performed in the transverse plane in all the patients.

The chemical-shift images were obtained by using both in-phase and opposed-phase T1-weighted gradient-echo sequences in all subjects. These images were acquired by using fast multiplanar spoiled gradient-echo sequences in a single breath-hold of 30-35 sec. TE for the in-phase images was of 4.6 msec and for the opposed-phase images, of 2.1 msec. Both in-phase and opposed-phase imaging was performed with the following parameters: TR, 155; flip angle, 90°; matrix, 256 × 192; NEX, 1; field of view, 40 cm; section thickness, 6 mm with a 1 mm intersection gap; and bandwidth, ± 62.50 kHz. We also performed T1- and T2-weighted FSE RT and fSPGR BH, with and without fat suppression.

### MR Image Analysis

The characterization of shape, size, contour and structure of the thymus gland is similar to the CT examinations. The current study was focused on the analysis of thymic gland signal intensity and chemical shift ratio (CSR).

The lesion was considered as homogeneous if it was composed of one signal intensity. If the lesion had heterogeneous signal intensity, we used the dominant signal intensity.

The signal intensity of the thymus gland was compared with that of the chest-wall muscle [**1**].

For quantitative evaluation we measured the signal intensities of the thymus gland and the chest-wall muscle on both in-phase and opposed-phase images.

Signal intensity measurements of the thymus and the paraspinal muscle were then obtained by using standard region-of-interest (ROI) electronic cursors, manually positioned at a slice level of the maximum axial surface of the thymus (area, 0.5-1.5 cm² for the thymus gland, respectively 1.1-2.5 cm² for the chest-wall muscle).

**Fig. 2:**
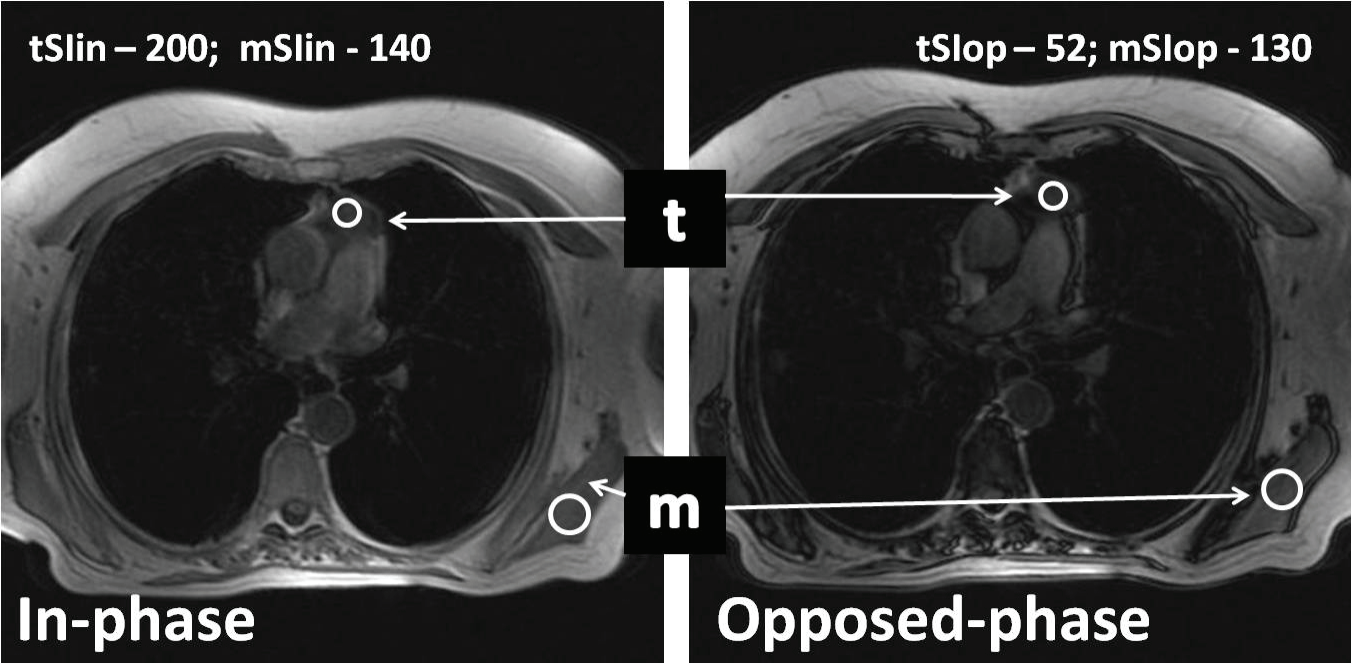
Signal intensity measurements of the thymus (t) and the chest-wall muscle (m) are obtained by using standard region-of-interest (ROI) electronic cursors, which are manually positioned. The chemical shift ratio (CSR), which was determined by comparing the signal intensity of the thymus gland (tSI) with that of the chest-wall muscle (mSI), both in phase (in) and opposed-phase (op) images, was calculated by using the following equation [**1,5**]: CSR = (tSIop/mSIop)/(tSIin/mSIin).

The chemical shift ratio (CSR), which was determined by comparing the signal intensity of the thymus gland (tSI) with that of the chest-wall muscle (mSI), both in phase (in) and opposed-phase (op) images, was calculated by using the following equation [**1,5**]: CSR = (tSIop/mSIop)/(tSIin/mSIin) (**Fig. 2**).

## Results 

**Fig. 3:**
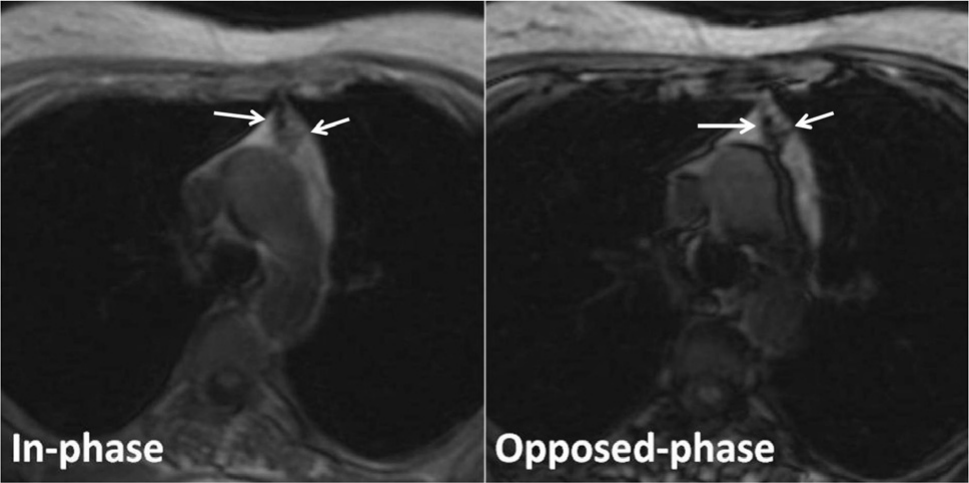
Thymic hyperplasia in a 40-year-old woman with myasthenia gravis. Transverse in-phase (155/4.6) and opposed-phase (155/2.1) gradient-echo T1-weighted MR images demonstrate an apparent decrease in signal intensity of the thymus gland on the opposed-phase image relative to the in-phase image (arrows). The CSR is of 0.761.

**Fig. 4:**
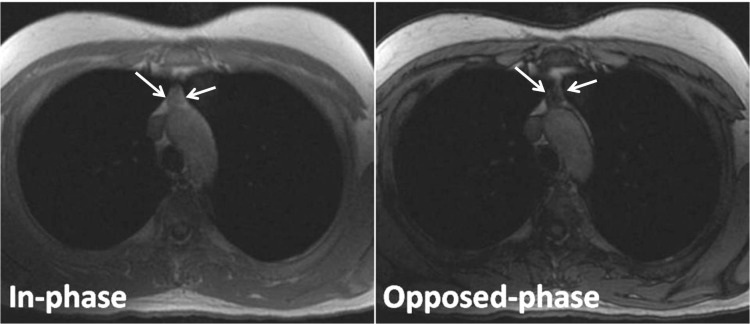
Thymic hyperplasia in a 26-year-old man with myasthenia gravis. Transverse in-phase (155/4.6) and opposed-phase (155/2.1) gradient-echo T1-weighted MR images demonstrate an apparent decrease in signal intensity of the thymus gland on the opposed-phase image relative to the in-phase image (arrows). The CSR is of 0.331.

In the hyperplasia group, the thymus gland showed diffuse enlargement without lobulation, and a homogeneous decrease in the signal intensity of the thymus gland on the opposed-phase image relative to the in-phase image in all patients (**Fig. 3,Fig. 4**).

**Fig. 5:**
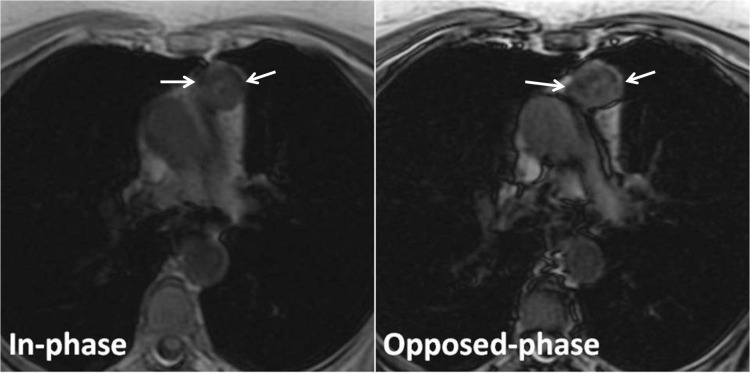
Thymoma in a 65-year-old woman with myasthenia gravis. Transverse in-phase (155/4.6) and opposed-phase (155/2.1) gradient-echo T1-weighted MR images demonstrate no change in signal intensity of the lesion on the opposed-phase image relative to the in-phase image (arrows). The CSR is of 1.010.

**Fig. 6:**
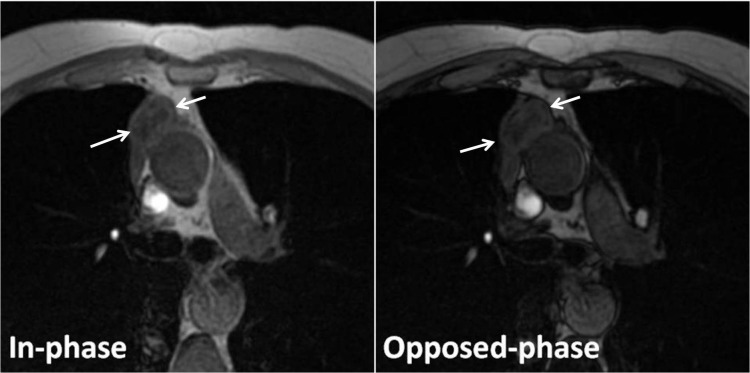
Thymoma in a 52-year-old man with myasthenia gravis. Transverse in-phase (155/4.6) and opposed-phase (155/2.1) gradient-echo T1-weighted MR images demonstrate no change in signal intensity of the lesion on the opposed-phase image relative to the in-phase image (arrows). The CSR is of 1.050.

The thymus gland was round or had an irregular shape in the tumor group, with no signal intensity loss in the thymus gland on the opposed-phase image, relative to the in-phase image in any patients (**Fig. 5,Fig. 6**).

**Fig. 7:**
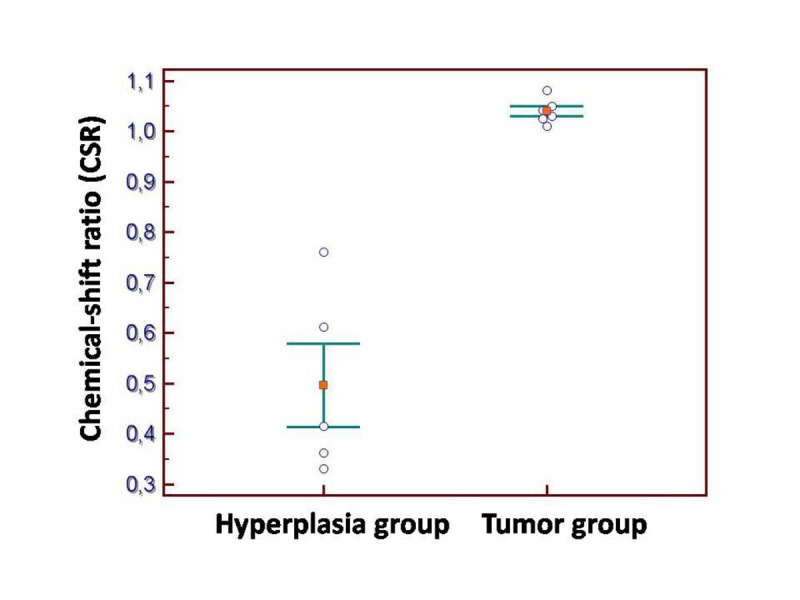
The CSR values are expressed as means ± standard deviations for the two groups. The mean CSR was of 0,4964 ± 0,1841 in the hyperplasia group and of 1,0398 ± 0,0244 in the tumor group. Statistically significant differences were seen between the hyperplasia and tumor groups (P=0,0028); there was no overlap in range.

The CSR values are expressed as means ± standard deviations for the two groups. To test between group differences (the hyperplasia group and the tumor group), the t test for unequal variance (Welch t test) was performed. The mean CSR was of 0,4964 ± 0,1841 in the hyperplasia group and of 1,0398 ± 0,0244 in the tumor group. Statistically significant differences were seen between the hyperplasia and tumor groups (P=0,0028); there was no overlap in range (**Fig. 7**).

## Discussion

The thymus achieves its maximal weight between 12 and 19 years; between 20 and 60 years, regression in size occurs, together with the replacement by adipose [**6**]. 

Therefore, the normal thymus contains various amounts of fat tissue, depending on age. The percentage of the total weight of the thymus that is fat tissue is of nearly 20% in the first decade of life and increases during the second decade, reaching 40%, late in the second decade [**5,7**]. 

Moore et al. reported that no distinct difference in the extent of fat replacement in the thymus was observed on CT between groups with normal thymic histology and those with microscopic hyperplasia in patients with MG [**5,8**]. 

Siegel et al. showed that the relative signal intensity of the normal thymus on T1-weighted images was slightly greater than that of muscle but less than that of fat. On T2-weighted images, they found that the signal intensity of the thymus was moderately greater than that of muscle but slightly less than or equal to that of fat [**5,9**]. Therefore, both the CT attenuation values and signal intensities on MR imaging of a normal thymus can overlap those of an abnormal thymus, especially in the first and second decades of life, when the gland has not been completely replaced by fat tissue [**5,9,10**]. 

Chemical shift MR imaging is much more sensitive than the other fat-suppressed MR sequences for the detection of microscopic fat within tissue, because it relies on the unique difference in resonance frequency between protons in water and those in triglyceride molecules [**1,11**]. This technique is widely used to diagnose lipid-containing lesions (lipoma, dermoid, teratoma, adrenal adenoma, focal fat within the liver, fatty liver and angiomyolipoma).

Sometimes, thymoma may show diffuse thymic enlargement at CT, and this can be incorrectly diagnosed as a hyperplastic thymus; in addition, hyperplastic thymus may appear at CT as a soft-tissue mass mimicking thymoma [**1**]. It can be challenging to differentiate the hyperplastic thymus from thymoma based on the morphologic assessment alone, especially in patients with MG [**1,12,13**].

Lymphoid thymic hyperplasia is most commonly associated with MG, being seen in up to 65% of the cases [**14**]. In patients with thymoma, the MG is present or appears in 30-50% of the cases [**15**]. 

Currently, the thymus is surgically resected in patients with MG because at least an improvement of clinical symptoms due to MG can be obtained, even if there is no associated thymoma [**15**]. Minimal invasive surgical techniques are not recommended in patients with thymic malignancies with high risk of local recurrence [**1**].

Chemical shift MR imaging can help differentiate between hyperplastic thymus and thymoma according to our study, based on the assessment of the CSR. CSR allows us to perform a quantitative assessment. The signal intensity of the thymus gland was compared with that of the chest-wall muscle on both in-phase and opposed-phase images.

All the patients in the hyperplasia group showed a homogeneous decrease in the signal intensity of the thymus gland on the opposed-phase image relative to the in-phase image. None of the patients in the tumor group showed a decrease in the signal intensity of the thymus gland at the chemical shift MR imaging. In addition, the mean CSR in the hyperplasia group was considerably lower than that in the tumor group, and there was no overlap in range.

We found recent data in literature that prove the usefulness of the chemical shift MR imaging to identify normal and hyperplastic thymus, by proving that normal fat infiltration and the differentiation of thymic hyperplasia from tumors of the thymus gland in patients with different pathological entities. 

Our study included only patients with myasthenia gravis, but an important limit of this study was the small number of patients.

A larger number of patients is necessary to clarify the utility of the chemical shift MR imaging for differentiating thymic hyperplasia from tumors of the thymus gland, in myasthenic patients.

## Conclusion

Occasionally, in the first and second decades of life, when the thymus gland has not been completely replaced by fat tissue, the CT attenuation values and signal intensities on MR imaging show what a normal or pathological thymus can overlap.

By using a qualitative comparison and by calculating the chemical-shift ratio between in-phase and opposed-phase gradient-echo images, particularly in patients with myasthenia gravis, it is possible to differentiate the hyperplastic thymus from the tumors of the thymus gland.
